# Does minimally invasive spine surgery reduce surgical site infection rates in the trauma patient? A Southeast Asian experience

**DOI:** 10.37796/2211-8039.1246

**Published:** 2022-03-01

**Authors:** ChengHan Wu, Kelvin Kah Ho Lor, Eugene Weiren Yang, Allan Shao Hui Ng

**Affiliations:** a Department of Orthopedic Surgery, Khoo Teck Puat Hospital, Singapore; b Department of General Surgery, Division of Neurosurgery, Khoo Teck Puat Hospital, Singapore

**Keywords:** Surgical site infection, Minimally invasive spine surgery, Trauma

## Abstract

**Introduction:**

The trauma patient has an increased susceptibility to postoperative surgical site infection (SSI). There is a lack of studies in the literature investigating the rates of SSI in minimally invasive spine (MIS) surgery for trauma patients with associated injuries, who also require surgical intervention for thoracolumbar fractures. We aim to investigate if MIS surgery for trauma patients reduces the incidence of SSI through a less invasive approach and smaller surgical incision.

**Methods:**

A case series of 30 trauma patients who underwent MIS surgery for thoracolumbar spine fractures at our center were followed up for a year. The primary outcome measured was the presence of a postoperative SSI. Subgroup analysis was performed to determine if there were specific factors that increase the risk of developing a SSI.

**Results:**

In total, 4 (13%) patients developed postoperative SSI out of which 1 was a deep infection (3%). Subgroup analysis of both patient and surgical factors did not demonstrate statistically significant results to suggest risk factors for SSI post-MIS surgery in our patient group.

**Conclusion:**

Our series of patients did not reflect a lower incidence of SSI with MIS surgery compared to incidences in the literature. This may suggest that the increased rates of SSI in the trauma patient may not be best addressed by a minimally invasive approach alone. A multidisciplinary approach that addresses other factors – such as prolonged recumbence and a compromised immunological state may yield improved results.

## 1. Introduction

Surgical site infection (SSI) after spinal surgery can lead to significant morbidity to the patient and an increased healthcare cost. SSI is devastating to the trauma patient, as it may necessitate long-term antibiotic administration, multiple procedures, and a prolonged hospital stay [1,2]. Varying incidences of SSI in spine surgery have been quoted in the literature, with some studies suggesting that patients suffering from traumatic spinal injuries have higher incidences of infection [3,4]. Existing studies in the literature suggest that minimally invasive spine (MIS) surgery for elective procedures can be a safe and effective option, with the added benefits of reducing infection rates, postoperative pain, and length of stay [5–7]. However, there is a paucity of studies in the literature with a focus on the incidence of SSI in trauma patients who require MIS surgery for thoracolumbar fractures.

Due to the higher energy nature of their injuries, the trauma patient undergoing surgery can be at a higher risk of developing SSI [8]. These patients generally need a longer length of stay in the hospital, allowing more opportunities for nosocomial wound colonization. Also, severe concomitant soft tissue injury following the index trauma may impede wound healing. Suboptimal hygiene from being recumbent for long periods and a compromised immunological state can also increase the risk of wound infection [9].

Most studies in the literature comparing the two approaches (open vs MIS) place an emphasis on the differences in clinical and radiographical outcomes, perioperative blood loss, and pain scores [10]. There are few studies dedicated to the study of post-surgical clinical outcomes in polytraumatic patients who also require thoracolumbar spine surgery. Our study aims to investigate if MIS techniques can result in a lower incidence of SSI compared to conventional open surgery in the trauma patient.

## 2. Material and methods

We retrospectively reviewed all trauma patients who underwent MIS surgery for thoracolumbar fractures in our institution over 3 years. The inclusion criteria were acute, traumatic thoracolumbar burst fractures that required surgical fixation in patients with concomitant injuries besides a spinal injury. Considerations for surgery included: Neurological deficits, kyphotic angle >20°, loss of vertebral body height (VBH) ≥50%, and retropulsion of bony fragments involving ≥50% of midsagittal diameter (MSD) of the fractured level. The exclusion criteria were [1]: Previous spinal instrumentation [2], Spinal tumors [3] Previous or coexisting spinal infection or systemic sepsis, and [4] Osteoporotic compression fractures.

After considering the above criteria, 30 patients were included in our study. All data were collected retrospectively from their medical case records or their electronic medical records with their consent.

All patients received a standard preoperative evaluation that included a full neurological examination and documentation of the ASIA score. Besides plain radiographs, all patients received a computed tomography (CT) scan of the thoracolumbar spine for surgical planning and assessment of the degree of deformity.

Prophylactic intravenous Cefazolin (dose titrated based on body weight) was given to all patients 30 minutes before incision as per standard institutional practice. In the event of a penicillin allergy, vancomycin was used. All MIS instrumentations were performed posteriorly via percutaneous stab incisions, through the deep fascia. Under fluoroscopic guidance, pedicle entry points were identified and cannulated using trephine needles. The needles were then advanced into the pedicle under radiographical guidance. Subsequent pedicle screws and rods instrumentation were performed as per standard practice. Distraction of the collapsed vertebral bodies to restore the height and correction of the kyphotic deformity were then performed with tubular retractors. The levels of instrumentation were decided based on the fracture pattern, location of the fracture, number of levels of fractured, bone quality, and age of the patient. Pedicle screws were avoided at the level of the fracture. The wound was closed in layers with Vicryl rapid 3–0. In patients with acute cord compression, a paramedian decompressive laminectomy was performed on the affected level before performing the screw fixation, using the tubular retractor system. No fusion was performed in all cases. A standard 2 more doses of antibiotics were administered post-operatively (for a total of 24 hours). All procedures were performed by an experienced, fellowship-trained consultant spine surgeon.

The primary outcome measured was the presence of postoperative SSI. Wound inspection was performed on postoperative day 3, on discharge, and during the patient’s outpatient visits at 2 weeks, 1 month, 3 months, 6 months, and 1 year postoperatively. When wound infection was evident, it is determined to be deep or superficial according to the Centers for Disease Control and Prevention (CDC) classification of surgical site infection (Table 1).

Subgroup analysis was performed to determine if the following factors increased the risk of developing SSI: Age, length of operation, length of stay, Injury severity score, concomitant injuries, ASA score, ASIA score, and the number of levels of fixation.

Statistical analyses of the data were performed using Statistical Package for the Social Sciences Version 12 for Windows (SPSS Inc, Chicago, IL, USA). An Independent *t*-test was performed for continuous data. Fisher’s exact test was performed for categorical data.

## 3. Results

30 patients met the study’s inclusion and exclusion criteria. The patients’ demographics are presented in Table 2. There were more females (63%) than males (37%). There are a variety of mechanisms of injury in these patients, namely, fall from height (15.0%), road traffic accident (30.0%), slip and fall (10.0%), and being hit by falling objects (10.0%). Within the population, 24 (80.0%), 5 (16.7%) and 1 (3.3%) had 1, 2 and 3 level of vertebral fractures, respectively. 23.3% of patients had 2, 13.3% of patients had 3, 50.0% of patients had 4, 10.0% of patients had 5 and 3.3% of the patients had >5 levels of vertebral bodies instrumented (Table 3).

Our study’s primary outcome measure was the incidence of postoperative SSI. All patients were followed up to a year post-surgery. Out of a total of 30 patients, 26 (86.7%) had no SSI while 4 (13.3%) of them suffered from SSI (Table 4). Amongst those with an infection, 1 (3.3%) had a deep surgical site infection and 3 (10.0%) had superficial infection. All cases involved the paramedian percutaneous wounds with the only case of deep infection also involving the midline laminectomy wound.

All 4 patients underwent surgical wound debridement. Amongst the patients with a superficial SSI, only 1 had a positive intra-operative culture (Methicillin-susceptible Staphylococcus Aureus). All 3 superficial SSI cases were treated successfully with intravenous and/or oral co-amoxiclav for a combined duration of 2 weeks. The fascial layer was noted to be involved in the patient who had a deep SSI. Implants were not removed as it was assessed to be not affected intraoperatively. Tissue cultures grew Pseudomonas Aeruginosa. The patient was treated initially with intravenous vancomycin and then oral ciprofloxacin subsequently. All SSI wounds healed successfully within 2 weeks and no further surgical debridement was required.

Subgroup analysis of the patient population was performed to determine if a certain patient or surgical factors predisposed them to develop surgical site infections. There were no statistically significant results that would suggest so (Table 5).

## 4. Discussion

SSI can lead to increased morbidity and mortality in patients as they may necessitate long-term antibiotic use, re-operation, and a prolonged hospital stay. The trauma patient has a higher risk of developing SSI compared to patients undergoing elective surgery [8]. MIS surgery has been demonstrated to have the benefit of reducing incidences of infection compared to open surgery in an elective environment [10,11]. However, there are still limited studies available in the literature to demonstrate the benefits of an MIS approach to thoracolumbar fractures in reducing SSI incidence in trauma patients with concomitant injuries.

Varying incidences of SSI have been observed for open surgery for thoracolumbar fractures in the literature. In Rechtine et al.’s study [12], 9 out of 117 patients (7.7%) with thoracic and lumbar fractures in their study suffered an SSI after undergoing open spine surgery. In a study by Cooper et al. [13], incidences of SSI for open fixation and instrumentation of thoracic and lumbar fractures were 3.0% and 7.9% respectively.

In contrast, incidences of SSI in patients with vertebral fractures undergoing MIS surgery in the literature seem to be lower. Palmisani et al.’s series of patients reported only 1 out of 51 patients (1.9%) who underwent MIS fixation for thoracolumbar fractures that developed a SSI requiring removal of instrumentation [14]. In a case series by Ni et al., they reported only 1 case of superficial infection out of the 36 patients (2.7%) that was treated successfully with antibiotics [15]. A comparative study between percutaneous and open pedicle screw fixations for thoracolumbar fractures by Lee et al. revealed no cases of SSI in the percutaneous group compared to 5.4% of the open group which required re-operation [16]. Lonjon and colleagues’ multi-centre prospective study on SSI in adult spinal trauma revealed a SSI rate of 4.2% in patients undergoing open surgery compared to 0% in patients who had MIS surgery [17].

Unfortunately, the varying methodologies of these studies render it difficult to draw direct comparisons to our patient cohort. In particular, the definition of SSI between these studies is not clearly defined, and it is uncertain if all SSI (superficial and deep) has been considered in their post-surgical outcomes. Also, polytraumatic patients are either excluded from their study population [17] or make up an uncertain or minority proportion of their study cohort.

Our study’s series of trauma patients who underwent MIS surgery had a comparatively higher incidence of SSI (both superficial and deep) (13.3%), compared to the papers above. This may be attributed to the unique characteristic of our patient population: [1] The patients included in our study are polytraumatic patients with concomitant injuries that may have reduced their mobility and/or resulted in a physiologically weaker immunological state. It is well established in the literature that polytraumatic patients are at higher risk of developing SSI [8,9]. [2] Also, being in a warmer, more humid tropical climate may have influenced higher rates of SSI post-operatively [18–20]. Our findings suggest that utilizing a smaller incision may not necessarily be the penultimate solution in reducing incidences of SSI in this group of trauma patients with concomitant thoracolumbar spine injuries. A multi-faceted approach addressing other issues in this unique population such as prolonged recumbency and a compromised immunological state may result in better results.

We also explored if there were any patient or surgical factors that could have predisposed the patients to develop post-operative SSI. However, there are no statistically significant results to suggest specific risk factors in our study population.

We acknowledge that there are a few limitations to our study. Firstly, the sample size is small which may predispose the findings to bias and limit the value of our subgroup analysis. Secondly, the study is an observational series that does not involve a comparison group. Hence, we can only draw comparisons from available incidences of SSI in patients who have undergone open or MIS fixation of traumatic thoracolumbar fractures in the literature. Future studies such as a larger prospective cohort study or randomized control trial will be helpful to better demonstrate the differences in incidences of SSI between open and MIS surgery in polytraumatic patients.

In conclusion, our series of Southeast Asian trauma patients did not reflect a lower incidence of SSI with MIS surgery compared to established rates in the literature. This may suggest that perhaps the reduction of SSI in this subset of patients may not be best addressed by a minimally invasive approach alone. Further investigation and the development of a multidisciplinary approach that addresses other factors – such as a prolonged recumbent state and the compromised immunological state of a polytraumatic patient may bring better results [21–23].

## Figures and Tables

**Table 1:** CDC definitions of surgical site infection.

Superficial incisional surgical site infection	Deep incisional surgical site infection
A superficial incisional SSI must meet the following criterion: Infection occurs within 30 days *and* involves only skin and subcutaneous tissue of the incision *and* patient has at least 1 of the following: Purulent drainage Organisms isolated from and ascptically obtained culture of fluid or tissue from the superficial incision At least 1 of the following signs or symptoms of infection: pain or tenderness, localised swelling, redness, or heal, and a superficial incision is deliberately opened by surgeon and is culture positive or not cultured. A culture negative finding does not meet this criterion Diagnosis of superficial SSI by the surgeon or attending physician	A deep incisional SSI must meet the following criterion: Infection occurs within 30 days if no implant is left in place or within 1 year if implant left in place and the infection appears to be related to the operative procedure *and* involves deep soft tissues (ie. Muscles and fascial layers) of the incision *and* patient has at least 1 of the following: Purulent drainage from the deep incision A deep incision spontaneously dehisces or is deliberately opened by a surgeon and is culture positive or not cultured when the patients has at least 1 of the following signs or symptoms: fever (>38). or localised pain or tenderness. A culture negative finding does not meet this criterion An abscess or other evidence of infection involving the deep incision is found on direct examination, during reoperation or by histopathological or radiological examination Diagnosis of superficial SSI by the surgeon or attending physician

**Table 2:** Patient demographics.

	Number of patients (%)
**Gender**	
Male	11 (36.6%)
Female	19 (63.3%)
**Age**	
<20	3 (10%)
21–30	10 (33.3%)
31–40	1 (3%)
41–50	6 (20%)
51–60	9 (30%)
>61	1 (3%)
**Comorbidities**	
Nil	20 (66%)
Cardiovascular disease	3 (10%)
Systemic disease	6 (20%)
Immunocompromised	1 (3%)

**Table 3:** Mechanism and degree of injury in the study population.

	Number of patients (%)
**Mechanism of injury**	
Fall from height	15 (50%)
Road traffic accident	9 (30%)
Slip and fall	3 (10%)
Hit by object	3 (10%)
**Number of levels of vertebral fractures**	
1	24 (80%)
2	5 (16.7%)
3	1 (3.3%)
**Number of vertebral levels fixed**	
2	7 (23.3%)
3	4 (13.3%)
4	15 (50.0%)
5	3 (10.0%)
>5	1 (3.3%)

**Table 4:** Incidence of SSI in the study population.

Surgical site infection	Number of patients (%)	Type	Number of patients (%)
Yes	4 (13.3)	Superficial	3 (10.0)
		Deep	1 (3.3)
No	26 (86.7)	N. A	N. A

**Table 5:** Subgroup analysis of patient and surgical factors predisposing to SSI.

	Surgical site infection (n = 4)	No surgical site infection (n = 26)	P value
Age	45.0 ± 11.4	38.4 ± 14.6	0.398
Length of operation (min)	158.8 ± 61.2	187.5 ± 81.1	0.504
Length of stay (days)	16.0 ± 9.2	24.2 ± 18.7	0.403
Injury severity score	19.7 ± 12.9	31.4 ± 15.1	0.467
ASA			0.743
1	1	12	
2	2	8	
3	0	2	
>3	0	0	
E	1	4	
ASIA score			0.628
A	1	2	
B	0	3	
C	0	3	
D	0	3	
E	3	15	
Levels of fixation			0.175
2	0	7	
3	2	2	
4	2	13	
5	0	3	
>5	0	1	
